# Trends in the Contribution of Genetic Susceptibility Loci to Hyperuricemia and Gout and Associated Novel Mechanisms

**DOI:** 10.3389/fcell.2022.937855

**Published:** 2022-06-23

**Authors:** Jianan Zhao, Shicheng Guo, Steven J. Schrodi, Dongyi He

**Affiliations:** ^1^ Department of Rheumatology, Shanghai Guanghua Hospital, Shanghai University of Traditional Chinese Medicine, Shanghai, China; ^2^ Guanghua Clinical Medical College, Shanghai University of Traditional Chinese Medicine, Shanghai, Shanghai, China; ^3^ Computation and Informatics in Biology and Medicine, University of WI-Madison, Madison, WI, United States; ^4^ Department of Medical Genetics, School of Medicine and Public Health, University of WI-Madison, Madison, WI, United States; ^5^ Arthritis Institute of Integrated Traditional and Western Medicine, Shanghai Chinese Medicine Research Institute, Shanghai, China; ^6^ Institute of Arthritis Research in Integrative Medicine, Shanghai Academy of Traditional Chinese Medicine, Shanghai, China

**Keywords:** hyperuricemia, gout, genetic susceptibility loci, novel mechanism, inflammation introduction

## Abstract

Hyperuricemia and gout are complex diseases mediated by genetic, epigenetic, and environmental exposure interactions. The incidence and medical burden of gout, an inflammatory arthritis caused by hyperuricemia, increase every year, significantly increasing the disease burden. Genetic factors play an essential role in the development of hyperuricemia and gout. Currently, the search on disease-associated genetic variants through large-scale genome-wide scans has primarily improved our understanding of this disease. However, most genome-wide association studies (GWASs) still focus on the basic level, whereas the biological mechanisms underlying the association between genetic variants and the disease are still far from well understood. Therefore, we summarized the latest hyperuricemia- and gout-associated genetic loci identified in the Global Biobank Meta-analysis Initiative (GBMI) and elucidated the comprehensive potential molecular mechanisms underlying the effects of these gene variants in hyperuricemia and gout based on genetic perspectives, in terms of mechanisms affecting uric acid excretion and reabsorption, lipid metabolism, glucose metabolism, and nod-like receptor pyrin domain 3 (NLRP3) inflammasome and inflammatory pathways. Finally, we summarized the potential effect of genetic variants on disease prognosis and drug efficacy. In conclusion, we expect that this summary will increase our understanding of the pathogenesis of hyperuricemia and gout, provide a theoretical basis for the innovative development of new clinical treatment options, and enhance the capabilities of precision medicine for hyperuricemia and gout treatment.

## Introduction

Gout is the leading cause of inflammatory arthritis in males. This is primarily due to multiple mechanisms resulting in the deposition of urate in the synovial fluid and other tissues to form monosodium urate crystals, which are further stimulated by inflammatory irritants, ultimately resulting in gout. The global prevalence of gout is approximately 0.1%–10%, and the incidence ranges from 0.3 to 6 cases per 1,000 person-years (Kuo et al., 2015; Liu et al., 2015; GBD, 2017). With a worldwide trend of an aging population, the medical disease burden of gout is increasing (Smith et al., 2014). Risk factors for hyperuricemia and gout include the use of medications (thiazides, cyclosporine, low-dose aspirin), insulin resistance, metabolic syndrome, obesity, renal insufficiency, abnormal blood pressure, purine-rich foods, alcohol, and sugary drinks (Neogi, 2011). The role of wine in gout may be contradictory, in any case, a retrospective study said individuals with established gout and pre-existing risk factors should limit all types of alcohol intake to prevent gout episodes (Nieradko-Iwanicka, 2021).

The main source of uric acid is the metabolism of purines and nucleotides in food produced in the liver and excreted by the intestines and kidneys (Köttgen et al., 2013a). Uric acid is reabsorbed and secreted into the proximal tubules of the kidneys, and urate transporter-1 (URAT1), glucose transporter 9 (GLUT9) (also known as GLUT9L), organic anion transporter 4 (OAT4), and organic anion transporter 4 (OAT10) are responsible for its reabsorption. TP-binding cassette superfamily G member 2 (ABCG2), adenosine triphosphate (ATP) binding cassette subfamily C member 4 (ABCC4), and organic anion transporter (NPT1 and NPT4) proteins mediate uric acid excretion. The balance between reabsorption and secretion is related to the homeostasis of uric acid. Otherwise, hyperuricemia and gout can occur. A high serum uric acid concentration is the primary risk factor for gout. Controlling the metabolism of uric acid in circulation at reasonable levels plays a vital role in preventing and improving gout (Chung and Kim, 2021). The progression from high blood uric acid levels to gout occurs in three main steps, hyperuricemia, the deposition of monosodium urate crystals, and inflammatory responses in the joints (Dalbeth et al., 2016). Toll-like receptor (TLR) and NLRP3 inflammasome activation and the associated inflammatory responses are critical factors in the progression of hyperuricemia to gout. This primarily involves activation of the downstream TLR4 and nuclear factor-κB (NF-kB) pathways, activation of the NLRP3 inflammasome, and production of interleukin (IL)-1β, which together regulate immune, metabolic, and inflammatory processes (Qing et al., 2013; McKinney et al., 2015; Rasheed et al., 2016).

Gene variants in related functional proteins can affect uric acid metabolism and inflammation *in vivo* (Reginato et al., 2012). Current research is focused on the heritability of uric acid-associated phenotypes, estimated to be around 40%–70%, implying a clear indication of the importance of its genetic role (Köttgen et al., 2013a). Commonly used drugs to treat acute gout attacks include nonsteroidal anti-inflammatory drugs (NSAIDs), colchicine, and glucocorticoids (Terkeltaub, 2003). Several biologically targeted agents have also been developed, such as IL-1/1β antagonists, anakinra, rilonacept, canakinumab (So et al., 2007; Terkeltaub et al., 2009; Neogi, 2010; So et al., 2010). In addition, patients with gout require a combination of long-term treatments to lower uric acid levels, such as allopurin, probenecid, and sulfinpyrazone (Terkeltaub, 2003). Although existing gout treatment drugs have achieved some efficacy, the multiple side effects and even poor drug response in some patients suggest that we should focus, at least in part, on the genetic mechanisms underlying hyperuricemia and gout to identify other effective and well-tolerated clinical treatment options. Large-scale genome-wide association studies have identified many risk loci (Tin et al., 2019a); however, the biological mechanisms underlying hyperuricemia and gout remain unclear. A meta-analysis of the Global Biobank Meta-analysis Initiative improved the understanding of this disease, as well as risk prediction, by integrating GWAS results from six major ancestral groups (African ancestry from African or mixed-race immigrants, mixed-race Americans, Central and South Asians, East Asians, Europeans, and Middle Easterners), while also providing insight into the underlying biology of the traits being studied by integrating gene and protein expression data, enabling the identification of disease-related genes and drug candidates (Zhou et al., 2021). This review discusses the mechanisms through which gene variants affect hyperuricemia and gout by searching *Pubmed* and *GBMI* (https://www.globalbiobankmeta.org/and http://results.globalbiobankmeta.org/) database. This review further explores and discusses the relationship between multiple biological agents and genetic variants and how they potentially affect gout and hyperuricemia to provide a theoretical reference for further clinical treatment options.

## Association Between Uric Acid Transporter-Related Gene Variants and Hyperuricemia and Gout

**FIGURE 1:**
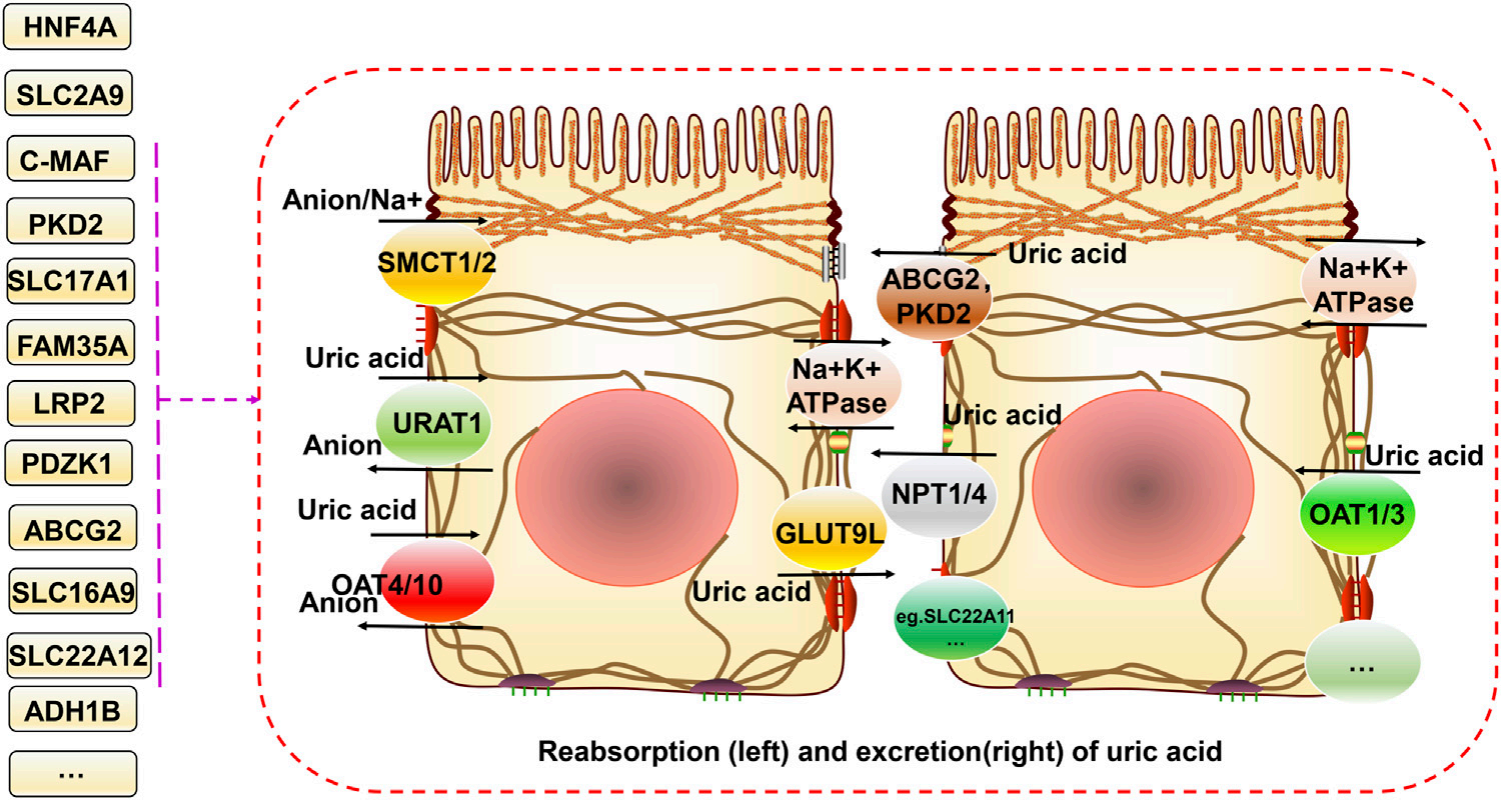
Relationship between gene variants and reabsorption and excretion of uric acid in hyperuricemia and gout. Uric acid is reabsorbed and secreted into the proximal tubules of the kidneys. URAT1, GLUT9 (also known as GLUT9L), OAT4, and OAT10 are responsible for its reabsorption. ABCG2, ABCC4, NPT1, and NPT4 mediate uric acid excretion. The balance between reabsorption and secretion is related to uric acid homeostasis. In addition, SMCT1/2 proteins can regulate ion concentrations both inside and outside the cell. Different gene variants have different effects on these processes.

A decline in kidney function is a vital cause of hyperuricemia and gout. As the kidney is the main organ that excretes uric acid, when this occurs, uric acid excretion by this organ is also reduced. The increase in uric acid in the blood aggravates hyperuricemia, negatively affecting each other and forming a vicious cycle (Asgari and Hilton, 2021). The proximal tubule excretes most uric acid, and when specific lesions occur there, dysfunction leads to low uric acid excretion, which is often associated with genetic variants of specific uric acid transport proteins. Pleiotropy in genetic variation can underlie the regulation of renal function, hyperuricemia, and gout (García-Nieto et al., 2022). For example, Mendelian randomization analysis can be used to analyze causality in confounding situations. Using this approach to determine the relationship between genetic variants regulating blood uric acid excretion in the kidney and renal function, it was found that the uric acid transporter genetic risk score (mainly comprising solute carrier family 2 member 9 (*SLC2A9*), ABCG2, solute carrier family 22 member 11 (*SLC22A11*), solute carrier family 17 member 1 (*SLC17A1*), and solute carrier family 22 member 12 (*SLC22A12*)) was positively associated with improved renal function in European Caucasian males. The uric acid transporter protein genetic risk score was used as an instrumental variable. Mendelian randomization for renal function using the two-stage least squares method to assess the effect of urate on renal function quantitatively (Hughes et al., 2014). In conclusion, it was found that the variant with the strongest effect on the protection of renal function was located in *SLC22A11* (Hughes et al., 2014). A meta-analysis further identified four gene loci (*SLC2A9*, *ABCG2*, *SLC22A12*, and *MAF BZIP transcription factor (MAF*)) associated with blood uric acid levels and renal function in an East Asian population (Okada et al., 2012). Another meta-analysis of GWASs on serum urea salt concentrations and gout in African Americans found genome-wide significance at three loci (*SLC2A9*, solute carrier family 2 member 12 (*SLC2A12),* and *SLC22A12*) (Tin et al., 2011). Previous genome-wide significant loci associated with serum urate levels, such as *SLC2A12*, were identified and validated in a meta-analysis by combining GWAS data from more than 14,000 individuals (Köttgen et al., 2013a). Similarly, *ABCG2*, *SLC2A9*, solute carrier family 16 member 9 (*SLC16A9)*, glucokinase (hexokinase 4) regulator *(GCKR)*, *SLC22A11*, *SLC22A12*, PDZ domain containing 1 (*PDZK1)*, and *SLC17A1* were found to be significantly associated with hyperuricemia and gout risk in Asian, native Hawaiian, and Pacific Islander populations estimated using the biospecimens repository at the University of Hawai’i (Alghubayshi et al., 2022). Most gout-related genetic studies have focused on this mechanism and have made some exciting discoveries, but there still could be a need to focus on this and explore it more extensively in the future (Figure 1).

### Hepatocyte Nuclear Factor 4 Alpha (HNF4A), Hepatocyte Nuclear Factor 4 Gamma (HNF4G), and PDZK1

*HNF4A* encodes a nuclear transcription factor that binds DNA and modulates the transcription of multiple genes, mainly in the form of homodimers. A missense variant in *HNF4A* (rs1800961) is probably the most likely leading and causal variant resulting in better transactivation of the promoter of the urate transporter protein-encoding gene *ABCG2* (Tin et al., 2019a). Additionally, HNF4A can also control gene expression in pancreatic islets, potentially further associating with uric acid and gout by affecting insulin secretion (Yoon et al., 2001). The inheritance and expression of different alleles of *HNF4A* might also have potential effects on renal function, but the exact mechanism remains unknown (Leask et al., 2020).

*PDZK1* primarily encodes a scaffolding protein containing the PDZ structural domain. It mediates the localization of cell surface proteins and is linked to cholesterol metabolism through the regulation of multiple receptors. HNF4A can also directly regulate *PDZK1*. The T-allele of *PDZK1* single-nucleotide polymorphism (SNP) (rs1967017) enhances HNF4A binding to the promoter of PDZK1, augmenting its expression, potentially increasing uric acid transport, and regulating uric acid homeostasis, as PDZK1 is a scaffolding protein for multiple transport proteins (Ketharnathan et al., 2018). A C-MAF BZIP transcription factor-encoding *(C-MAF)* SNP (rs889472) might also be associated with gout susceptibility by affecting uric acid metabolism (Higashino et al., 2018), and part of the mechanism could be related to regulation of the transcription factor HNF4A (Leask et al., 2018). MAF/c-MAF is mainly expressed in the proximal tubules of the kidney and is a critical factor for maintaining differentiation and functional integrity (Imaki et al., 2004; Tsuchiya et al., 2015). Its b-ZIP structure can form dimers with other b-ZIP proteins and bind to DNA as transcription factors to regulate the functions of various organs, such as the kidney and pancreas (Yang and Cvekl, 2007; Tsuchiya et al., 2015). Leask et al. summarized the genetic mechanisms underlying the detailed regulation of uric acid levels mediated by *MAF* variants, mainly involving the proximal signal cis-expression quantitative trait loci (*cis-eQTL*) of *MAF* (controls the expression of MAF transcriptional regulator RNA *(MAFTRR*)) and the distal signal *cis-eQTL* (controls the expression of LINC01229). The MAFTRR lncRNA region binds to the MAF promoter and recruits the histone imprint H3K27me3 to repress MAF transcription, whereas the removal of both LINC01229 and *MAFTRR* promotes MAF expression (Leask and Merriman, 2021).

*HNF4G* also encodes a transcription factor involved in the positive regulation of transcription by RNA polymerase II. It has a lower transcriptional activation potential than that of HNF4A. An *HNF4G* SNP (rs2941484) can increase gout susceptibility in the Chinese population and mainly affects serum uric acid concentration and gout risk in men (Dong et al., 2017). In Chinese Han men, the TT genotype of the *HNF4G* rs2941484 may represent a gender-specific genetic marker of hyperuricemia. The distribution frequency of TT and CC+CT alleles in hyperuricemic and normokalemic males differed considerably (*p* = 0.011) in the rs2941484 recessive model (Chen et al., 2017). miR-34a can regulate HNF4G to control the survival, proliferation, and invasion of bladder cancer cells (Sun et al., 2015) and might bind to endogenous fatty acids to regulate fatty acid metabolic pathways affecting gout (Wisely et al., 2002).

### ABCG2 and Polycystin 2 (PKD2)

ABCG2 is a multispecific heterotrimeric and endogenous transporter protein expressed mainly in the kidney, liver, and gastrointestinal tract that affects drug metabolism and plays a key role in uric acid excretion. Its variants can lead to destabilization of the nucleotide-binding structural domain of ABCG2, resulting in its reduced expression and dysfunction, leading to the inadequate renal excretion of urate, causing hyperuricemia and gout (Wong et al., 2016). *ABCG2* variants (rs2231142) are variants associated with gout and an increased frequency of erythema (Onuora, 2020). Individuals carrying the *ABCG2* SNP (rs2231142) have a nearly 2-fold increased susceptibility to gout (Lee et al., 2019), and alcohol consumption independently increases the risk of gout stones in the Han Chinese population in Taiwan (Tu et al., 2014). The *alpha kinase 1* variant in combination with *the ABCG2 SNP* (rs2231142), the SLC2A9 SNP (rs1014290), or *the SLC22A12 SNP* (rs475688 and rs3825016) is linked to gout in the recessive model (Tu et al., 2018). *ABCG2* SNP (rs2231142) significantly increased the risk of gout in Asians (dominant model: OR = 2.64, 95% CI = 2.04–3.43, *p* = 0.02 for heterogeneity; recessive model: OR = 3.19, 95% CI = 2.56–3.97, *p* = 0.28 for heterogeneity; co-dominant model: OR = 1.37, 95% CI = 1.18–1.59, *p* = 0.09 for heterogeneity) as well as other populations (dominant model: OR = 1.85, 95% CI = 1.20–2.85, *p* < 0.0001 for heterogeneity; recessive model: OR = 3.78, 95% CI = 2.28–6.27, *p* = 0.19 for heterogeneity; co-dominant model: OR = 1.48, 95% CI = 1.26–1.74, *p* = 0.19 for heterogeneity) (Li et al., 2015a). The *ABCG2* SNP (rs72552713) also significantly increased the risk of gout in Asians (dominant model: OR = 3.87, 95% CI = 2.07–7.24, *p* = 0.06 for heterogeneity) (Li et al., 2015a). *ABCG2* and *PKD2* were found to have epistatic interactions, and two SNP pairs (rs2728121:rs1481012 and rs2728121:rs2231137) were mainly identified as associated with the serum urate concentration or risk of hyperuricemia (Dong et al., 2020). ABCG2 variants might affect disease progression through inflammatory pathways, in addition to lowering uric acid excretion. The knockdown of ABCG2 in endothelial cells leads to higher IL-8 release, which further leads to inflammation (Chen et al., 2018). ABCG2 deficiency in hepatocytes leads to mitochondrial dysfunction and dynamics. Owing to increased intracellular protoporphyrin IX/DRP-1-mediated mitochondrial fission, abnormal protein function results in aggregate formation, leading to excessive reactive oxygen species activation of the NLRP3 inflammasome, which plays a role in the development of gout (Lin et al., 2013). Mitochondrial dysfunction can induce the NLRP3 inflammasome in gout to promote IL-1β and inflammation (Gosling et al., 2018). In addition, monosodium urate crystals also disrupt proteasomal degradation, leading to increased P62 expression, impaired cellular autophagy, and the inability to clear dysfunctional proteins, thus leading to aggregates formation. An *ABCG2* SNP (rs2231142) enhances this autophagic impairment, diminishes the formation of neutrophil extracellular traps, and aggravates gout via the overactive release of the NLRP3 inflammasome and IL-1β. Neutrophil extracellular traps can degrade cytokines and chemokines to limit inflammation (Luciani et al., 2010; Shi et al., 2012; Choe et al., 2014; Schauer et al., 2014). *PKD2* is localized near *ABCG2* and encodes a urate transporter protein. *PKD2* variants in autosomal dominant polycystic kidney disease result in PKD2 transporter dysfunction and elevated serum urate concentrations, which are associated with hyperuricemia and gout (Mejías et al., 1989; Puig et al., 1993). A transcript assay revealed that *PDK2* and *ABCG2* gene expression levels are positively correlated; thus, the regulators of PDK2 interact with ABCG2 to indirectly influence gout incidence (Dong et al., 2020).

### SLC16A9, SLC17A1, and Shieldin Complex Subunit 2 (FAM35A)

A *SLC16A9* SNP (rs2242206) can affect the function of its encoded monocarboxylate transporter 9 (MCT9) protein, resulting in inadequate urate excretion in the kidney (Kolz et al., 2009; Nakayama et al., 2013). SLC2A9 is expressed in the liver, kidney, and bone cells and transports various substances, including urates and sugars. The SNP rs734553 alters protein affinity to increase the risk of hyperuricemia and gout (Yi et al., 2018).

*SLC17A1* encodes the NPT1 protein. The SNP rs1183201 appears to have a protective effect against diseases by enhancing urate excretion and transport (Kolz et al., 2009). A meta-analysis of GWASs on serum uric acid and gout in 28,283 Caucasian individuals found genome-wide significance for the *SLC17A1* SNP with serum urate levels (Yang et al., 2010).

*FAM35A* variants are associated with gout and hyperuricemia via a mechanism that might involve a reduction in uric acid excretion during renal excretion (Nakayama et al., 2017). FAM35A encodes a DNA repair protein expressed mainly in the distal tubules of the kidney and has not been directly linked to uric acid metabolism in functional assays. Therefore, there might be other indirect mechanisms and the potential involvement of kidney function in the regulation of uric acid excretion (Nakayama et al., 2017; García-Nieto et al., 2022).

### SLC22A12

*SLC22A12* encodes the transporter protein URAT1, which is primarily responsible for urate reabsorption following urine filtration. Tin et al. identified 97 rare variants of *SLC22A12*, of which functional validation of *p. Trp325*, *p. Cys405*, and *p. Met467* variants revealed that they cause loss of function of the encoded protein affecting serum uric acid levels. Individuals carrying *SLC22A12* variants have a lower risk of developing gout (Tin et al., 2018). Linkage disequilibrium between *SLC22A12* and *SLC22A11* might be associated with uric acid in Caucasian individuals (Yang et al., 2010). Novel G65W variants of *SLC22A12* (rs12800450) are characterized as functional alleles with an approximately 6–10-fold greater effect on uric acid than that observed for common variants in *SLC22A12* (Tin et al., 2011). Existing drugs have been developed to target URAT1, such as probenecid and benzbromarone. In addition, a new URAT1 inhibitor for the treatment of chronic gout, lesinurad (Zurampic®; RDEA594), was approved in the United States and Europe in 2016 (Miner et al., 2016). However, lesinurad alone appears to impair renal function and should be used in combination with xanthine oxidase inhibitors, and recipients should be closely monitored for renal function (Narang and Dalbeth, 2018). The alcohol dehydrogenase 1B (Class I), beta polypeptide (*ADH1B*) SNP (rs129984) might increase the NADH/NAD ratio to promote lactate production by facilitating ethanol conversion to highly reactive acetaldehyde, thereby increasing uric acid reabsorption in synergy with the *SLC22A12*-encoded transporter protein URAT1 (Lieber et al., 1962; Edenberg, 2007; Macgregor et al., 2009; Sandoval-Plata et al., 2021).

### Solute Carrier Family 22 Member 6 (SCL22A6)

*SLC22A6* primarily encodes organic anion transporter 1 (OAT1) involved in eliminating endogenous and exogenous organic anions from the kidney. Tanner et al. identified multiple SNPs in *SLC22A6* associated with hyperuricemia, including rs3017670, rs2276300, rs4149171, and rs4149170. Strong association studies with gout have been performed; however, there is potential evidence linking it to gout (Tanner et al., 2017). Granados et al. found altered tryptophan metabolite profiles in SLC22A6-knockout mice, including several gut microbiota metabolites that are thought to be deleterious for chronic kidney disease. Probenecid, a gout treatment drug, elevates the levels of circulating tryptophan metabolites. Different variants affect the ability of OAT1 to regulate tryptophan metabolism, thus potentially causing gout. Therefore, based on the relationship between OAT1 and tryptophan metabolism, it might be a potential future direction for targets of drug development (Granados et al., 2021). Liu et al. also demonstrated that OAT1 is associated with various metabolic processes, including the tricarboxylic acid cycle, tryptophan metabolism, and other amino acids, fatty acids, and prostaglandins (Liu et al., 2016).

### SLC2A9

*SLC2A9* mainly encodes the GLUT9 protein. The missense variants (rs16890979) of *SLC2A9* showed an association with uric acid and gout (Dehghan et al., 2008). On the one hand, *SLC2A9* is related to regulation by the transcription factor HNF4A. HNF4A overexpression enhances the activity of SLC2A9. The mRNA expression levels of *HNF4A* and *SLC2A9* are significantly correlated, and there is an interaction between them (Prestin et al., 2014). The contribution of the coding sequence variants of *SLC2A9* to overall uric acid metabolism is still unknown because of the presence of linkage disequilibrium and heterogeneity, but 24 annotated nonsynonymous variants have been identified (Reginato et al., 2012). The effects of variants in *SLC2A9* (Val253Ile, and Arg265His) are also inconsistent based on studies on gout and hyperuricemia, and further studies are required (Hollis-Moffatt et al., 2009; Tu et al., 2010; Urano et al., 2010; Reginato et al., 2012). On the other hand, SLC2A9 can exchange uric acid with glucose and fructose, which are involved in gluconeogenesis. This may also have a potential impact on hyperuricemia and gout (Batt et al., 2014).

### EFFECT OF GENE VARIANTS RELATED TO GLUCOLIPID METABOLISM ON HYPERURICEMIA AND GOUT

Multiple metabolic factors, including abnormalities in glucose regulation, lipid levels, obesity, and arterial hypertension, are associated with primary gout and hyperuricemia (González-Senac et al., 2014). The glycolytic pathway leads to increased serum uric acid levels through various mechanisms. In addition, insulin resistance and high blood glucose levels can directly affect uric acid clearance in the kidneys (Padova et al., 1964). Hyperinsulinemia increases urate reabsorption in the kidney and decreases renal uric acid and sodium excretion, and this effect can also occur at sites other than the proximal tubule (Quinones Galvan et al., 1995; Maaten et al., 1997). Studies have demonstrated that a high intake of fructose or other high-calorie foods can dramatically increase serum uric acid levels beyond what the body can typically handle, resulting in urate deposition with a severe disruption in hepatocyte metabolism. The rapid intake of fructose can also cause an increase in blood lactate, probably via a mechanism involving blockage of the gluconeogenic pathway caused by the inhibition of glucose-phosphate isomerase mediated by fructose 1-phosphate, leading to the excessive production of lactic acid in hepatocytes (Perheentupa and Raivio, 1967). In addition, fructose phosphorylation in the liver can increase serum uric acid levels by interacting with aldolase B, ATP, and adenosine monophosphate deaminase 2 (AMPD2) (Lanaspa et al., 2011). Dyslipidemia, insulin resistance, hyperuricemia, and gout are interrelated (Schmidt et al., 1996). Excessive alcohol intake, but not including wine, has been shown to increase serum uric acid levels in several studies (Choi and Curhan, 2004; Yu et al., 2008). Some alcohols, such as beer, contain high levels of purines, and excessive intake can increase uric acid synthesis, leading to hyperuricemia and resistance to some of the antioxidant components of the plasma (van der Gaag et al., 2000; Nishioka et al., 2002). The specific underlying mechanism might involve the degradation of adenosine triphosphate to monophosphate during alcohol metabolism, thereby increasing adenosine and uric acid synthesis. The oxidation of alcohol (ethanol) increases blood lactate, further decreases uric acid excretion, and potentially affects lipid metabolism, thereby increasing the risk of hyperuricemia and gout (Nakamura et al., 2012). Multiple gene variants are associated with glucose metabolism and are potentially associated with hyperuricemia and gout.

### GCKR, MLX Interacting Protein (MLXIP), and MLX Interacting Protein-Like (MLXIPL)

*GCKR* encodes the GCKR subfamily of proteins that are regulatory proteins that inhibit glucokinase in the liver and pancreatic islet cells by binding noncovalently with the enzyme to form inactive complexes. *GCKR* variants (rs1260326) are missense variants that serve as possible candidate causal variants for which the leucine allele leads to increased glucokinase GCK activity, resulting in increased glycolytic flux, which facilitates hepatic glucose metabolism (Beer et al., 2009). A *GCKR* SNP (rs780094) is strongly associated with gout in Polynesian, European, Japanese, and Chinese populations (Wang et al., 2012; Köttgen et al., 2013a; Urano et al., 2013). In the recessive model, *GCKR* SNP (rs780094) was shown to be associated with the risk of hyperuricemia in men in the Uyghur population of Xinjiang in China (*p* = 0.015, OR = 1.311) (Wang et al., 2018). GCKR and NFAT5 are associated with glucose metabolism or the insulin response, and GCKR increases the metabolites that cause gout-related factors through glycolysis (Köttgen et al., 2013a; Rasheed et al., 2017). *MLXIP* encodes a protein that forms a heterodimer with MAX dimerization protein. It regulates the genes that moderate cellular glucose levels. *MLXIPL* encodes a Myc/Max/Mad superfamily basic helix-loop-helix leucine zipper transcription factor that forms a heterodimeric complex. That binds and activates the carbohydrate response element-binding protein motif within the triglyceride synthesis gene promoter in a glucose-dependent manner. *MLXIP and MLXIPL* variants can also correlate with serum urate concentrations (Boocock et al., 2020). MLXIPL is primarily associated with cellular carbohydrate metabolism and glycolytic processes. It is directly responsible for regulating glucose flux, interpreted as the pentose phosphate pathway producing ribose 5-phosphate, an essential precursor of *de novo* purine synthesis, and is involved in the production of uric acid (Köttgen et al., 2013a). In addition, the overproduction of lactate affects the transmembrane transport of urate, leading to impaired clearance of urate by the kidney (Luo et al., 2005; Tong et al., 2009; Levine and Puzio-Kuter, 2010).

### Patatin-Like Phospholipase Domain-Containing 3 (PNPLA3) and Insulin Like Growth Factor 1 Receptor (IGF1R)

*PNPLA3* encodes an active lipase that hydrolyzes various lipids and is associated with oxidative stress (Huang et al., 2011). A *PNPLA3* SNP (rs738409) is associated with hyperuricemia in a Japanese population (Nakatochi et al., 2019a). The rs738409-G allele was found to be associated with a reduced risk of gout in phenome-wide association studies (Diogo et al., 2018). This study indicated that the *PNPLA3* SNP (rs738409) enhances susceptibility to metabolism-related fatty liver disease (MAFLD) and is involved in the pathology of liver fibrosis (Kawaguchi et al., 2018; Namjou et al., 2019). In the recessive model, the *PNPLA3* SNP (rs738409) was associated with NAFLD in different ethnic groups in China: Han (OR = 1.84, 95% CI: 1.03–3.27, *p* = 0.036), Uyghur (OR = 2.25, 95% CI: 1.23–4.09, *p* = 0.006) (Zhang et al., 2014). *IGF1R* encodes the insulin-like growth factor I receptor. This receptor binds insulin-like growth factor with a high affinity. It has tyrosine kinase activity. The insulin-like growth factor I receptor plays a critical role in transformation events. *IGF1R* SNPs (rs12908437, rs659854, rs1291127, and rs4966024) might correlate with blood uric acid levels by affecting the body mass index (BMI) (Park et al., 2021). An abnormal BMI is indicative of abnormal lipid metabolism, and plasma uric acid is a powerful antioxidant (Ames et al., 1981). Thus, *PNPLA3* and *IGF1R* variants might be linked to hyperuricemia and gout by affecting lipid metabolism and oxidative stress.

### APOBEC1 Complementation Factor (A1CF)

*A1CF* encodes a protein that may primarily act as an RNA binding subunit and be involved in RNA editing or processing. Rasheed et al. found that both a *GCKR* SNP (rs780094) and *A1CF* SNP (rs10821905) interact with alcohol exposure to increase the risk of gout in a European population under alcohol exposure conditions, suggesting that the involvement of GCKR and AICF in alcohol metabolism promotes the development of gout (Rasheed et al., 2017). The *A1CF* SNP has been previously associated with hyperuricemia (Köttgen et al., 2013b). Makoto et al. further investigated the association between the *A1CF* SNP (rs10821905) and gout in Japanese individuals. They found that it was significantly associated with elevated serum uric acid and gout via a mechanism that might involve the regulation of dyslipidemia and uric acid metabolism (Kawaguchi et al., 2021). Further investigation of the mechanism of interaction between alcohol and AICF could suggest that the metabolite acetate of alcohol (ethanol) leads to the increased production of diacylglycerol and further activates protein kinase C and AICF phosphorylation in the nucleus. This leads to the increased production of apolipoprotein B (ApoB)-48 and decreased production of ApoB-100 at the transcriptional level, further causing increased free fatty acid production from very-low-density lipoproteins/triglycerides, which stimulates downstream TLR, the NLRP3 inflammasome, and IL-1β to activate the inflammatory response and produce more monosodium urate crystals (Joosten et al., 2010; Jump et al., 2013; Rasheed et al., 2017).

### Lipoprotein Receptor-Related Protein 2 (LRP2)

*LRP2* encodes an endocytic receptor protein, low-density lipoprotein-related protein 2, which is associated with multiple ligands such as ApoB, lipoprotein lipase, and lactoferrin. It is expressed in numerous tissues, such as proximal renal tubules (Christensen and Birn, 2002). *LRP2* SNPs (rs2390793, rs2544390, and rs16856823) are associated with blood uric acid (Kamatani et al., 2010; Kanai et al., 2018; Nakatochi et al., 2019b; Tin et al., 2019b) and increased gout susceptibility in Japanese (Akashi et al., 2020) and Chinese populations (Dong et al., 2015). However, its variants might lead to renal tubular dysfunction, affecting the renal reabsorption of uric acid (Kamatani et al., 2010; Kanai et al., 2018; Nakatochi et al., 2019b; Tin et al., 2019b). In contrast, rs2544390 was shown to have a non-additive interaction effect with alcohol consumption (beer or spirits), which can increase the risk of serum urate accumulation and gout in alcohol drinkers (Rasheed et al., 2013). However, there are additional contradictory results showing that LRP2 is not associated with gout susceptibility (Nakayama et al., 2014). LRP2 can also regulate the activity of lipoprotein lipase to modulate lipid metabolism, which is associated with uric acid metabolism (Rasheed et al., 2013). Additional experiments are needed to clarify the potential biological mechanisms and links between LRP2 and hyperuricemia and gout.

### NLRP3 INFLAMMASOME AND INFLAMMATION-ASSOCIATED GENES PROMOTE THE PROGRESSION OF HYPERURICEMIA TO GOUT

**FIGURE 2:**
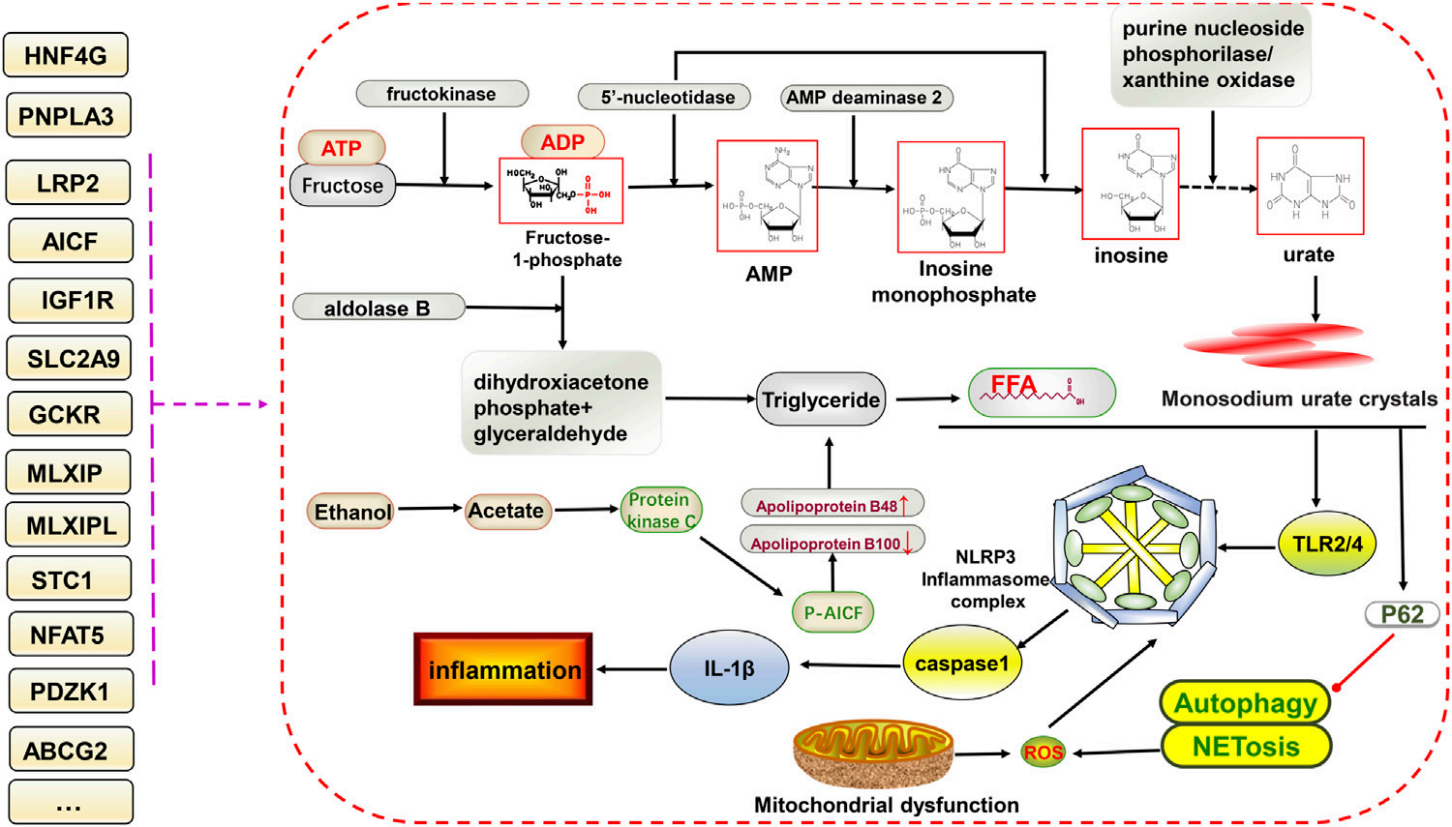
Potential relationship between gene variants and glucolipid metabolism and NLRP3 inflammasome-mediated inflammatory pathways in hyperuricemia and gout. The ingestion of fructose increases uric acid formation via the gluconeogenic pathway. In addition, triglycerides are also produced, increasing free fatty acid contents via the lipid metabolic pathway. Free fatty acids and monosodium urate crystals together stimulate downstream TLR2/4 and NLRP3 inflammasome formation, facilitate the of release IL-1β, and inhibit P62-mediated activation of autophagy and NETosis, ultimately promoting inflammation. Gene variants have different effects on different processes.

Pyroptosis, involving the NLRP3 inflammasome, can lead to cell destruction and the release of the pro-inflammatory factors IL-18 and IL-1β, thus promoting inflammation, which has been discussed in rheumatoid arthritis and MAFLD. Both have similarities to gouty arthritis in terms of disease mechanisms (Zhao et al., 2021a; Zhao et al., 2021b). As mentioned earlier, the excessive deposition of uric acid leads to the appearance of monosodium urate crystals, which are stimulated by the NLRP3 inflammasome and inflammatory factors to progress further toward inflammation. Many genetic variants could be involved in this **(** Figure 2 **)**.

### IGF1R

IGF1R might be associated with activation of the NLRP3 inflammasome in gout. Spadaro et al. found that macrophages lacking the IGF1R have reduced NLRP3 activation and a controlled inflammatory response (Spadaro et al., 2016). Liang et al. found that IGF1R primarily regulates vascular homeostasis and precise endothelial functions and that IGF1R deficiency impairs endothelial function in experimental mice and increases the degree of fibrosis in renal disease, which is associated with a poor wound healing response owing to repeated irritation from inflammation (Liang et al., 2015). Thus, both studies suggest that IGF1R variants might influence gout by regulating inflammation. In addition, a *IGF1R* SNP (rs7193778) and *PDZK1* SNP (rs112129861) could interact with each other, further enriching our understanding of the genetic and biological mechanisms underlying uric acid accumulation and gout (Fernández-Torres et al., 2019).

### Stanniocalcin 1 (STC1)

*STC1* encodes stanniocalcin-1, a glycoprotein that plays a role in multiple biological responses, including bone development, angiogenesis, and inflammatory responses (Yeung et al., 2012). Studies have reported that *STC1* is associated with elevated serum uric acid levels (Köttgen et al., 2013b). An *STC1* SNP (rs17786744) might cause the crystalline precipitation of sodium urate to trigger the inflammatory process, further exacerbating cartilage damage and promoting knee osteoarthritis, which could be associated with the inflammatory response in gouty arthritis. In addition, an interaction between an *STC1* SNP (rs17786744) and *GCKR* SNP (rs1260326) synergistically promotes crystalline precipitation with urate-promoting gout (Fernández-Torres et al., 2019).

## The Association Between Genetic Variants Involved in Other Mechanisms and Hyperuricemia and Gout

Various factors, such as coffee intake, tryptophan metabolism, B-cell development and activation, and sex hormones, are interlinked with genetic variants that play a role in hyperuricemia and gout. Hutton et al. found a negative association between coffee intake and gout. ABCG2, GCKR, MLXIPL, and cytochrome P450 family 1 subfamily A member 2 (CYP1A2) are variants associated with coffee consumption habits, and GCKR and ABCG2 are associated with low coffee intake and a high gout risk. Coffee consumption habits indirectly affect the association between gene variants and gout. In contrast, the direct effect of these gene variants on gout is still possible through other mechanisms, as described previously herein (Hutton et al., 2018). Evidence from studies involving genetic variants associated with other mechanisms is relatively scarce, and further research is needed in the future. Therefore, in this section, we summarize briefly the association of other mechanisms with gout, including sleep rhythm, immune response and B-cell activation (hypocretin receptor 2 (*HCRTR2*), cytokine-dependent hematopoietic cell linker (*CLNK*), guanine nucleotide-binding protein a-stimulating polypeptide (*GNAS*)), sex hormones (breast cancer-amplified sequence 3 (*BCAS3*)).

### HCRTR2, CLNK and GNAS

The protein encoded by *HCRTR2* is a G protein-coupled receptor involved in the regulation of feeding. The encoded proteins bind to orexin A and orexin B. A *HCRTR2* SNP (rs4715517), a variant associated with serum uric acid, appears to be specific to Asian populations with significantly higher allele frequencies than those in European populations. Differences in allele frequencies might contribute to interethnic differences in serum uric acid levels (Park et al., 2021). HCRTR2 is mainly involved in the sleep rhythm of the body (Lane et al., 2017; Dashti et al., 2019). With the accelerated pace of life in modern society, irregular sleep affects the immune system and the function of multiple organs, including the kidneys and liver. Therefore, genes regulating sleep rhythms might be potentially associated with hyperuricemia and gout. CLNK, a member of the SLP76 family, plays an essential role in integrating immunotyrosine-based activation motif-bearing receptors and integrins and is a positive regulator of immune response signaling (Yu et al., 2001; Wu and Koretzky, 2004). The allele “G" of *CLNK* SNP (rs2041215 and rs1686947) was identified as susceptibility genes for gout in the Chinese population by using dominant model (OR 1.66; 95% CI 1.04–2.63; *p* = 0.031) (OR 2.19; 95% CI 1.38–3.46; *p* = 0.001) and additive model (OR 1.39; 95% CI 1.00–1.93; *p* = 0.049) (OR 1.67; 95% CI 1.19–2.32; *p* = 0.003), respectively (Jin et al., 2015). A *CLNK* SNP (rs16869924) within the established *SLC2A9* gout-associated locus was shown to increase the risk of gout in Polynesian and Chinese Tibetan individuals, genetically independent on the *SLC2A9* association signal (Lan et al., 2016; Ji et al., 2021). It is hypothesized that CLNK mainly regulates B-cell development and activation and co-mediates the formation of immune complexes through the STAT signaling pathway to promote gout, as suggested be a combination of related studies (Siniachenko et al., 1984; Wang et al., 2002; Marrero et al., 2006). *GNAS* encodes GSa protein, which activates downstream cyclic AMP (cAMP) production and promotes signaling (Turan and Bastepe, 2015; Tafaj and Jüppner, 2017). *GNAS* variants predispose patients to an abnormal synovial environment and the deposition of uric acid crystals, promoting the formation of gout and related osteoarthritis (Rhyu and Bhat, 2021).

### BCAS3

*BCAS3* encodes proteins that are associated with several functions, such as angiogenesis, activation and recruitment of cell division cycle 42, reorganization of the actin cytoskeleton at the leading edge, regulation of cell polarity, the endothelial cell migration, filopodia formation, estrogen receptor response, and autophagy.

Rs11653176 in *BCAS3* is significantly associated with uric acid levels and gout in Japanese and Chinese Han populations (Li et al., 2015b; Sakiyama et al., 2018). BCAS3 can activate estrogen receptor alpha (Sakiyama et al., 2018). Studies have shown that sex hormones can affect uric acid levels (Adamopoulos et al., 1977). Postmenopausal women might have elevated uric acid levels owing to a decrease in estrogen, especially estradiol, because estrogen is more effective in promoting urate clearance by the kidneys (Hak and Choi, 2008). Similarly, the effects of some gene variants on serum uric acid levels and gout appear to be sex-specific. For example, variants in *SLC2A9* and *ABCG2*, which are associated with urate concentrations, are sex-specific (Köttgen et al., 2013a). An *SLC16A9* SNP (rs12356193) was found to be weakly associated with gout but strongly associated with blood uric acid and showed a sex-specific difference (Köttgen et al., 2013a). It is thus possible that sex hormones primarily contribute to sex differences in disease or drug efficacy.

### Gene Variants as Potential Diagnostic Markers of Drug Efficacy and Prognosis

A case-control association study of gout in Chinese populations revealed that *CLNK* SNPs (rs2041215 and rs1686947) are associated with various clinicopathological parameters and might have potential as diagnostic and prognostic markers for patients with gout (Jin et al., 2015). Patients with gout carrying an *ABCG2* SNP (rs2231142) respond poorly to allopurine therapy (Wen et al., 2015; Roberts et al., 2017; Wallace et al., 2018). A GWAS and polygenic risk scores in patients with asymptomatic hyperuricemia and gout revealed that *ABCG2* (rs2231142, rs13120400, and rs7672194), *SLC2A9* (rs16890979 and rs16891234), *SLC22A11* (rs2078267), *GCKR* (rs1260326), matrix extracellular phosphoglycoprotein (*MEPE*) (rs114580333), protein phosphatase, Mg2+/Mn2+ dependent 1 K-divergent transcript (*PPM1K-DT*) (rs4693211, rs28793136, and rs1545207), LO*C105377323* (rs114791459), and alcohol dehydrogenase 1B (*Class I*)*, beta polypeptide* (*ADH1B*) (rs1229984) SNPs can be used as markers of asymptomatic hyperuricemia to identify transition predictors (Sandoval-Plata et al., 2021). However, little research has been conducted on genetic variants as markers to predict disease progression and drug efficacy, as serum uric acid levels can effectively predict the risk of gout. However, this might provide more relevant results that could be uncovered through in-depth studies in the future.

## Conclusion

Gout is a form of arthritis that damages patients’ physical and mental health and causes severe pain during acute attacks. Identifying individuals at risk in the early stages of the disease is essential to prevent and reduce hyperuricemia and gout and to provide pharmacological and lifestyle interventions to better treat patients with clinically diagnosed gout. The identification of genetic variants might help in disease prevention and intervention. Many GWASs have performed to uncover loci related to hyperuricemia and gout, mostly linking it to uric acid transporter proteins, such as the widely studied URAT1 and GLUT9. Some drugs have been used as targets for drug development (see Table 1). We also summarize the latest clinical trials of these genes, and some of these were conducted in the context of gout, which is certainly instructive. Although some trials were not investigated in the context of gout, they have some informative implications for the clinical management of gout, which urgently needs to be studied in depth in the context of gout in the future (see Table2). Currently, the most elucidated is the effect of variants in the uric acid transporter protein gene on hyperuricemia and gout. We aim to increase our understanding of the genetic mechanisms behind the disease by adding descriptions of other genes of potential clinical value. All of these genes are undoubtedly promising and essential. Associations between genetic variants and traits are often located in regions of strong linkage disequilibrium and aided by eQTL analysis and fine localization studies, these can be exploited to identify true causal variants of gout in complex genetic backgrounds. Genetic-related issues in multiple disease contexts still need attention and elucidation, such as disease-specific genetic variants in different ethnic backgrounds, genetic variants based on sex differences, rare and low frequency variants, functional polymorphisms in genetic susceptibility genes, and epigenetic mechanisms. With the rapid development of modern molecular biotechnologies and multi-omics techniques, these issues require further clarification. In addition, attention should be paid to the interconnection between hyperuricemia/gout and other diseases, such as metabolic syndrome and cardiovascular diseases, as well as the role of genetic factors in these diseases. Elucidating these genetic issues will contribute to the improvement of clinical outcomes and precision medicine.

**TABLE 1:** Gene variants associated with hyperuricemia and gout.

Items	SNPs and Its Potential Impact	Molecular Mechanisms and Associations	Ref
*HNF4A*	rs1800961 (+)	Has a stronger activating effect on ABCG2	Tin et al. (2019a)
*PDZK1*	rs1967017 (-) rs112129861 (+)	Enhances binding to HNF4A to increase uric acid transport and interacts with IGF1R to regulate the inflammatory response	(Ketharnathan et al., 2018; Fernández-Torres et al., 2019)
*C-MAF*	rs889472 (un)	Can interact with HNF4A and is associated with gout susceptibility	Higashino et al. (2018)
*ABCG2*	rs2231142 (+) rs2231137 (+) rs1481012 (+) rs13120400 (+) rs7672194 (+)	Associated with early-onset gout, erythema, and gout stone appearance Variants destabilize the nucleotide-binding structural domain of ABCG2 and inflammatory responses Also interacts with the SNP of PKD2 and serves as a diagnostic and prognostic marker	(Tu et al., 2014; Wong et al., 2016; Dong et al., 2020; Onuora, 2020; Sandoval-Plata et al., 2021)
*PKD2*	rs2728121 (+)	Interacts with the SNP of *ABCG2* to increase the risk of gout and increase urate concentration	(Mejías et al., 1989; Puig et al., 1993; Dong et al., 2020)
*SLC2A9*	rs734553 (+) rs16890979 (un) rs16891234 (+)	Alters protein affinity to increase the risk of hyperuricemia and gout and can be used as a diagnostic and prognostic marker	(Yi et al., 2018; Sandoval-Plata et al., 2021)
*SLC17A1*	rs1183201 (-)	Protects against disease by enhancing urate excretion and transport and is associated with glucose metabolism	Kolz et al. (2009)
*FAM35A*	rs7903456 (+)	Reduces the excretion of uric acid in the kidneys	Nakayama et al. (2017)
*LRP2*	rs2390793 (+) rs2544390 (+) rs16856823 (+)	Mainly affects the renal reabsorption of uric acid, alcohol, and lipid metabolism	(Kamatani et al., 2010; Rasheed et al., 2013; Kanai et al., 2018; Nakatochi et al., 2019b; Tin et al., 2019b)
*SLC22A12*	rs150255373 (-) rs563239942 (-) rs200104135 (-) rs528619562 (-) rs12800450 (-)	Protective factor against gout that functions by altering protein function	Tin et al. (2018)
*ADH1B*	rs129984 (+)	It mainly affects the renal reabsorption of uric acid and acts synergistically with transporter protein URAT1 and can be used to predict the transition from asymptomatic hyperuricemia to gout	(Lieber et al., 1962; Edenberg, 2007; Macgregor et al., 2009; Sandoval-Plata et al., 2021)
*HNF4G*	rs2941484 (+)	Associated with gout by regulating endogenous fatty acid metabolism	Wisely et al. (2002)
*PNPLA3*	rs738409 (-)	Affects gout susceptibility by influencing lipid metabolism and oxidative stress processes	Diogo et al. (2018)
*IGF1R*	rs12908437 (un) rs659854 (un) rs659854 (un) rs1291127 (un) rs4966024 (un) rs7193778 (un)	Affects gout susceptibility by influencing lipid metabolism and oxidative stress processes and modulates the inflammatory response by interacting with PDZK1	Park et al. (2021)
*GCKR*	rs780094 (+) rs1260326 (+)	Regulates uric acid levels by modulating glucolipid metabolism, promotes an inflammatory response by interacting with STC1, and can be used as a diagnostic and prognostic marker	(Köttgen et al., 2013b; Fernández-Torres et al., 2019; Sandoval-Plata et al., 2021)
*A1CF*	rs10821905 (+)	Regulates uric acid levels by modulating dyslipidemia and alcohol metabolism	Köttgen et al. (2013b)
*MLXIP*	rs7953704 (un)	Transcription factor that might regulate serum uric acid through the pentose phosphate pathway	Boocock et al. (2020)
*MLXIPL*	rs1178977 (un)	Responsible for regulating glucose flux and potentially affects the renal clearance of urate	(Hutton et al., 2018; Boocock et al., 2020)
*STC1*	rs17786744 (+)	Promotes the precipitation of monosodium urate crystals to activate the inflammatory response	(Köttgen et al., 2013b; Fernández-Torres et al., 2019)
*CLNK*	rs16869924 (+) rs2041215 (+) rs1686947 (+)	Regulates B-cell development and activation and mediates the formation of immune complexes through the STAT signaling pathway and might serve as a diagnostic and prognostic marker	(Siniachenko et al., 1984; Wang et al., 2002; Marrero et al., 2006; Jin et al., 2015)
*SLC22A6*	rs3017670 (un) rs2276300 (un) rs4149171 (un) rs4149170 (un)	Might be associated with the regulation of tryptophan metabolism	Granados et al. (2021)
*BCAS3*	rs11653176 (+)	Activates estrogen receptor alpha to regulate sex hormone levels affecting uric acid levels	Sakiyama et al. (2018)
*SLC16A9*	rs12356193 (un)	Might be related to sex hormone regulation	Köttgen et al. (2013a)
*HCRTR2*	rs4715517 (un)	Might affect the immune system by regulating sleep rhythms	(Lane et al., 2017; Dashti et al., 2019)
*SLC22A11*	rs2078267 (+)	Can be used to predict the transition from asymptomatic hyperuricemia to gout	Sandoval-Plata et al. (2021)
*MEPE*	rs114580333 (+)	Can be used to predict the transition from asymptomatic hyperuricemia to gout	Sandoval-Plata et al. (2021)
*PPM1K-DT*	rs4693211 (+) rs28793136 (+) rs1545207 (+)	Can be used to predict the transition from asymptomatic hyperuricemia to gout	Sandoval-Plata et al. (2021)
*LOC105377323*	rs114791459 (+)	Can be used to predict the transition from asymptomatic hyperuricemia to gout	Sandoval-Plata et al. (2021)

(+), The SNP promotes hyperuricemia or gout (-), The SNP inhibits hyperuricemia or gout (un), The SNP has unknown or contradictory effects on hyperuricemia or gout.

**TABLE 2:** Gene tests in clinical trials.

Items	ClinicalTrials.gov identifier	Condition or Disease	Intervention/Treatment	Aims
*HNF4A*	NCT01181505	Genotype guided (HNF4a), healthy subjects	Tolterodine	To study the effect of the *HNF-4A* G60D variant on the CYP2D6 activity *in vivo*
	NCT04239586	Maturity onset diabetes in the young (MODY)	Sulfonylurea	To detect the association of the *HNF4A* variant with insulin secretion in MODY.
*ABCG2*	NCT03710395	Hypertensive breastfeeding women	Nifedipine	The present study aimed to evaluate the effect of *ABCG2* c.421C>A on nifedipine breast milk/plasma concentration ratio in hypertensive breastfeeding women
	NCT04410965	Multiple sclerosis	Teriflunomide	To evaluate the relationship between *ABCG2* mutation (rs2231142) and teriflunomide exposure
	NCT04608344	Rheumatoid arthritis	Atorvastatin, pravastatin, rosuvastatin, filgotinib	To evaluate the effect of filgotinib on a mixed organic anion transporting polypeptide/cytochrome P450 3A (OATP/CYP3A), OATP/breast cancer resistance protein (BCRP), and OATP substrates
*PKD2*	NCT02112136	Autosomal dominant polycystic kidney disease (ADPKD)	Blood collection	To identify families with ADPKD, characterize the phenotype, and screen for mutations in *PKD2* genes
*SLC22A12*	NCT04040907	Gout	XNW3009, placebo	XNW3009 is a small molecule hURAT1 inhibitor. The study investigates the safety, tolerability, pharmacokinetics, and pharmacodynamics of XNW3009
	NCT02815839	Gout, hyperuricemia	SHR4640, placebo	SHR4640 is a URAT1 inhibitor. The study assesses the safety, tolerance, food effect, and pharmacokinetic and pharmacodynamic properties of single-dose administration of SHR4640 in healthy volunteers
	NCT01883167	Healthy	RDEA3170, febuxostat, placebo	To evaluate the potential pharmacokinetic and pharmacodynamic interaction between the xanthine oxidase inhibitor febuxostat and the investigational URAT1 inhibitor RDEA3170
	NCT03316131	Asymptomatic hyperuricemia	RDEA3170, febuxostat, dapagliflozin, placebo	To assess the effect of intensive uric acid lowering therapy with RDEA3170, febuxostat, and dapagliflozin on urinary excretion of uric acid, in asymptomatic hyperuricemic patients
*GCKR*	NCT01023750	Hypertriglyceridemia, insulin resistance	Fenofibrate	To study the pretreatment genotyping at *GCKR* loci and response to fenofibrate therapy
*PNPLA3*	NCT04640324	Non-alcoholic fatty liver disease, insulin resistance	Nutraceutical therapy	To explore the effect of 303 mg of silybin-phospholipids complex, 10 mg of vitamin D, and 15 mg of vitamin E twice a day for 6 months in NAFLD patients carrying *PNPLA3*-rs738409, *TM6SF2*-rs58542926, and *MBOAT7*-rs641738 genetic variants
	NCT04483947	Non-alcoholic steatohepatitis (NASH)	AZD2693, placebo	AZD2693 is a PNPLA3 inhibitor. This study is intended to investigate the safety and tolerability, pharmacokinetics, and pharmacodynamics of AZD2693 in NASH patients carrying *PNPLA3*-rs738409
*GNAS*	NCT04671719	Fibrous dysplasia, albright syndrome, adult children hypoparathyroidism hyperparathyroidism pseudo hypoparathyroidism	blood sample	To study the full spectrum of PTH and GNAS pathologies
*SLC22A6*	NCT02743260	Healthy	Pitavastatin, metformin, digoxin, Adefovir sitagliptin	To establish *in vivo* phenotyping procedures for organic anionic transporter polypeptide 1B1 (OATP1B1), organic cation transporters 1 and 2 (OCT1/2), multidrug and toxic compound extrusion transporters 1 and 2, kidney splice variant (MATE1/2K), organic anion transporters 1 and 3 (OAT1/3), and p-glycoprotein (P-gp) transporters via a cocktail approach

## Glossary

GWAS: genome-wide association studies
GBMI: Global Biobank Meta-analysis Initiative
URAT1: urate transporter-1
GLUT9: glucose transporter 9
OAT4: organic anion transporter 4
OAT10: organic anion transporter 4
ABCG2: TP-binding cassette superfamily G member 2
ABCC4: ATP Binding Cassette Subfamily C Member 4
ATP: adenosine triphosphate
TLR: Toll-like receptor
NLRP3: nod-like receptor pyrin domain 3
NF-kB: nuclear factor-κB
IL: interleukin
NSAIDs: non-steroidal anti-inflammation drugs
SLC2A9: solute carrier family 2 member 9
SLC22A11: solute carrier family 22 member 11
SLC17A1: solute carrier family 17 member 1
SLC22A12: solute carrier family 22 member 12
MAF: MAF BZIP transcription factor
SLC2A12: solute carrier family 2 member 12
SLC16A9: solute carrier family 16 member 9
GCKR: glucokinase (hexokinase 4) regulator
PDZK1: PDZ domain-containing 1
HNF4A: hepatocyte nuclear factor 4 alpha
SNP: single-nucleotide polymorphism
C-MAF: C-MAF BZIP transcription factor
cis-eQTL: cis-expression quantitative trait loci
MAFTRR: MAF transcriptional regulator RNA
HNF4G: hepatocyte nuclear factor 4 gamma
PKD2: polycystin 2
ADH1B: alcohol dehydrogenase 1B (Class I), beta polypeptide
MCT9: monocarboxylate transporter 9
FAM35A: shieldin complex subunit 2
AMPD2: adenosine monophosphate deaminase 2
MLXIP: MLX interacting protein
MLXIPL: MLX interacting protein-like
PNPLA3: patatin-like phospholipase domain containing 3
MAFLD: metabolism-related fatty liver disease
IGF1R: insulin like growth factor 1 receptor
BMI: body mass index
A1CF: APOBEC1 complementation factor
ApoB: apolipoprotein B
LRP2: lipoprotein receptor-related protein 2
STC1: stanniocalcin 1
CYP1A2: cytochrome P450 family 1 subfamily A member 2
HCRTR2: hypocretin receptor 2
CLNK: cytokine-dependent hematopoietic cell linker
GNAS: guanine nucleotide-binding protein a-stimulating polypeptide
SCL22A6: solute carrier family 22 member 6
BCAS3: breast cancer-amplified sequence 3
cAMP: cyclic AMP
OAT1: organic anion transporter 1
MEPE: matrix extracellular phosphoglycoprotein
PPM1K-DT: protein phosphatase, Mg2+/Mn2+ dependent 1K- divergent transcript
ADH1B: alcohol dehydrogenase 1B (Class I), beta polypeptide
